# The Tumor Necrosis Factor Alpha and Interleukin 6 Auto-paracrine Signaling Loop Controls Mycobacterium avium Infection via Induction of IRF1/IRG1 in Human Primary Macrophages

**DOI:** 10.1128/mBio.02121-21

**Published:** 2021-10-05

**Authors:** Alexandre Gidon, Claire Louet, Lisa Marie Røst, Per Bruheim, Trude Helen Flo

**Affiliations:** a Centre of Molecular Inflammation Research, Norwegian University of Science and Technology, Trondheim, Norway; b Department of Clinical and Molecular Medicine, Faculty of Medicine and Health Sciences, Norwegian University of Science and Technology, Trondheim, Norway; c Department of Biotechnology and Food Science, Faculty of Natural Sciences, Norwegian University of Science and Technology, Trondheim, Norway; d Department of Infection, St. Olavs Hospital, Trondheim, Norway; Washington University School of Medicine in St. Louis

**Keywords:** *Mycobacterium avium* infection, innate immunity, human primary macrophages, itaconate, TNF-α, IL-6, IRF1, IRG1

## Abstract

Macrophages sense and respond to pathogens by induction of antimicrobial and inflammatory programs to alert other immune cells and eliminate the infectious threat. We have previously identified the transcription factor IRF1 to be consistently activated in macrophages during Mycobacterium avium infection, but its precise role during infection is not clear. Here, we show that tumor necrosis factor alpha (TNF-α) and interleukin 6 (IL-6) autocrine/paracrine signaling contributes to controlling the intracellular growth of M. avium in human primary macrophages through activation of IRF1 nuclear translocation and expression of IRG1, a mitochondrial enzyme that produces the antimicrobial metabolite itaconate. Small interfering RNA (siRNA)-mediated knockdown of IRF1 or IRG1 increased the mycobacterial load, whereas exogenously provided itaconate was bacteriostatic at high concentrations. While the overall level of endogenous itaconate was low in M. avium-infected macrophages, the repositioning of mitochondria to M. avium phagosomes suggests a mechanism by which itaconate can be delivered directly to M. avium phagosomes in sufficient quantities to inhibit growth. Using mRNA hybridization, we further show that uninfected bystander cells actively contribute to the resolution of infection by producing IL-6 and TNF-α, which, via paracrine signaling, activate IRF1/IRG1 and strengthen the antimicrobial activity of infected macrophages. This mechanism contributes to the understanding of why patients on anti-inflammatory treatment, e.g., with tocilizumab or infliximab, can be more susceptible to mycobacterial disease.

## INTRODUCTION

Mycobacterium avium is an environmental pathogen that causes opportunistic infection in people with underlying lung disease or that are immunocompromised, e.g., from immunosuppressive treatment, such as neutralization of tumor necrosis factor alpha (TNF-α) (1–3). Like other pathogenic mycobacteria, M. avium infects primarily macrophages and can cause persistent infection by subverting intracellular trafficking, degradation, and antimicrobial activities (4–8).

We have previously reported that M. avium infection strongly induces the expression and nuclear translocation of the transcription factor interferon (IFN) regulatory factor 1 (IRF1) in human primary macrophages, with delayed kinetics compared to that of nuclear factor κB (NF-κB) (6, 7). IRF1 activation was reduced upon silencing of Toll-like receptors 7 and 8 (TLR7/8), their adaptor (MyD88), and the trafficking chaperone Unc93B1 or by inhibition of the inhibitor of NF-κB kinase β (IKKβ), but the role of IRF1 in mycobacterial host defenses was not clarified in these studies. Others have also shown that *Irf1* expression is induced in mycobacterial infection (9, 10) and contributes to the control of mouse infections with Mycobacterium bovis BCG (11), Mycobacterium tuberculosis (12), or a virulent strain of M. avium (9). All together, these studies call for a better understanding of the role of IRF1 in antimycobacterial immunity.

IRF1 is potently induced by interferons, in particular, IFN-γ, but other cytokines, including TNF-α, interleukin 1β (IL-1β), and IL-6, as well as engagement of pattern recognition receptors (PRRs), have been shown to activate IRF1 in cell- and context-dependent manners (13–20). IRF1 is differentially involved in the transcriptional regulation of immune responses and the development of lymphoid immune cells and function (18), in part by regulating chromatin accessibility (21). It was first identified as a positive regulator of IFN-β and later shown to be a mediator of interferon responses involved in driving the expression of the inducible nitric oxide synthase gene (*iNOS*), *IL-12*, *STAT*s, guanylate binding protein genes (*GBP*s), immune-responsive genes (*IRG*s), and type I and/or type III IFNs in a stimulus- and cell-dependent manner (13–19). IRF1 is thus strongly connected to interferon responses and antiviral immunity (18). IRF1 activity is also linked to metabolism and to the tricarboxylic acid (TCA) cycle in particular. Tallam and colleagues have shown that IRF1 controls IRG1 (22), a mitochondrial enzyme which is responsible for the production of itaconate, a metabolite with antimicrobial (23–25) and immunoregulatory (26–32) activities. Furthermore, Nair and colleagues have shown that IRG1 diminishes pathology from excessive neutrophil infiltration and that *Irg1^−/−^* mice rapidly succumb to M. tuberculosis infection (33). Here, we demonstrate that M. avium, directly via TLRs and indirectly via autocrine/paracrine TNF-α and IL-6 signaling, activates IRF1 nuclear translocation and the expression of IRG1 in infected and uninfected bystander human macrophages. IRG1 controls mycobacterial growth, possibly through directed delivery of itaconate to M. avium phagosomes.

## RESULTS

### TNF-α and IL-6 autocrine/paracrine signaling enhances IRF1 activation in M. avium infection.

**FIG 1:**
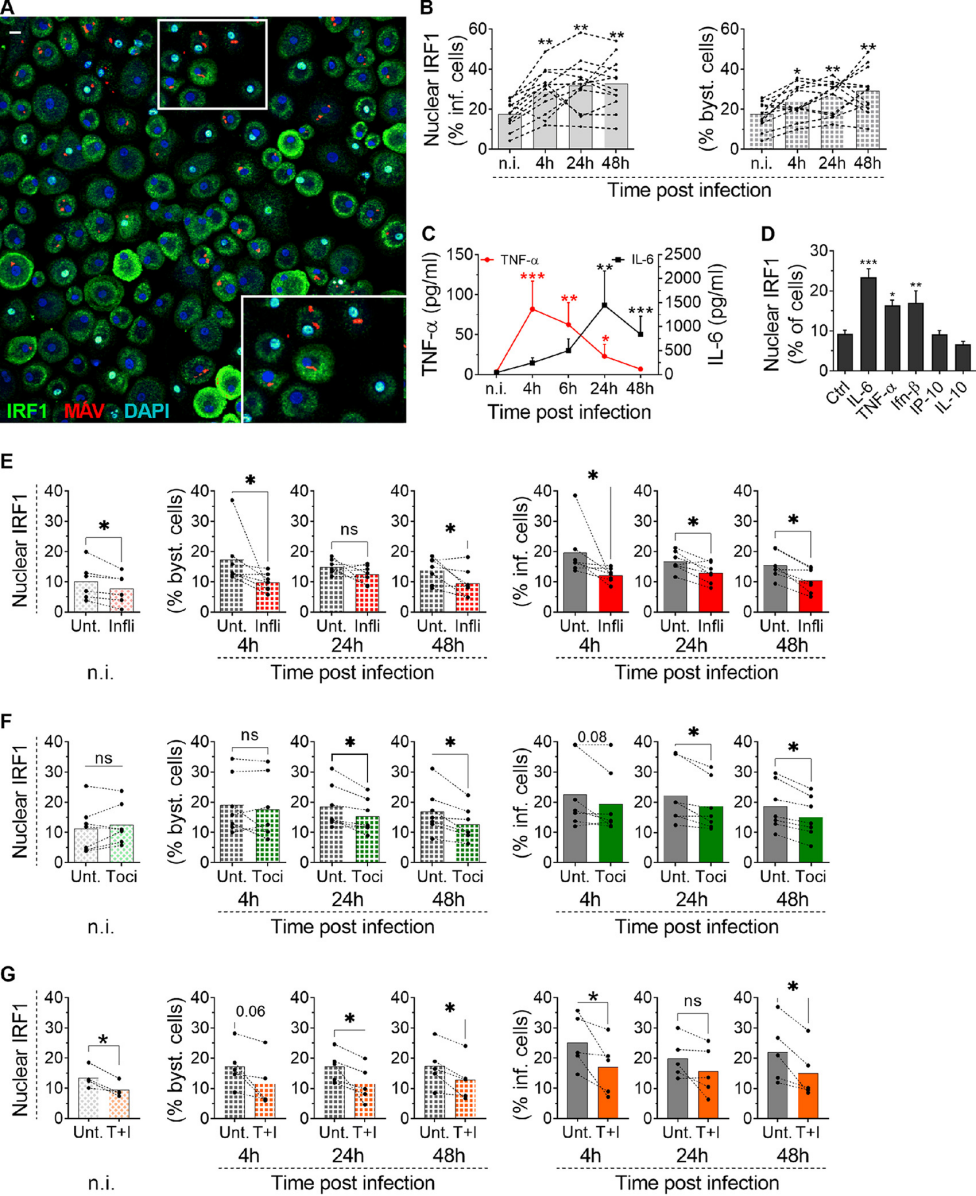
TNF-α and IL-6 contribute to IRF1 activation in M. avium-infected macrophages. Human MDMs were infected with live M. avium-DsRed for 10 min, followed by a chase of up to 48 h. (A) The nuclear translocation of IRF1 was analyzed by confocal microscopy at the indicated time points using anti-IRF1 antibodies, together with a nuclear stain (Hoechst dye). MAV, *M. avium*; DAPI, 4′,6-diamidino-2-phenylindole. (B) Quantification of IRF1 nuclear translocation (left graph, infected [inf.] macrophages; right graph, uninfected bystander [byst.] macrophages). Traces represent individual donors, and bar charts represent averages for 9 donors for each time point (>600 cells per time point and per donor). (C) TNF-α (red trace) and IL-6 (black trace) were measured in the supernatants using a multiplex ELISA. Traces represent the means of results for 11 independent donors. n.i., noninfected. (D) Human MDMs were treated with 25 nM rIL-6, rTNF-α, rIFN-β, rIP-10, or rIL-10 for 4 h. The nuclear translocation of IRF1 was analyzed by confocal microscopy. Bar charts represent averages of results for 3 different donors (>500 cells per condition and per donor). Ctrl, control. (E to G) Human MDMs were infected with live M. avium-DsRed for 10 min, followed by a chase of up to 48 h in the presence of 2 μg/ml infliximab (Infli) (E, red), 2 μg/ml tocilizumab (Toci) (F, green), or 2 μg/ml tocilizumab and infliximab (T+I) (G, orange). Unt., untreated. The nuclear translocation of IRF1 was analyzed by confocal microscopy at the indicated time points. Dots represent the average value per donor (>500 cells per time point), and bar charts represent averages of results for different donors (at least 5). Bar charts to the left are noninfected cells (light-colored pattern), charts in the middle are uninfected bystander cells (dark-colored pattern), and charts to the right are infected cells (solid color). (B, C) *P* values between results for noninfected cells and different time points were calculated using the nonparametric ANOVA test; (D to G) *P* values between the control and treated conditions for each time point were calculated using the nonparametric paired Wilcoxon signed-rank test. *, *P < *0.05; **, *P < *0.01; ***, *P < *0.001; ns, not significant. The scale bar represents 10 μm.

To gain further insight into the role of IRF1 during M. avium infection in macrophages, we asked how IRF1 is activated. Primary human monocyte-derived macrophages (MDMs) were infected with M. avium for 4, 24, and 48 h, and IRF1 activation was assessed by monitoring its nuclear localization (Fig. 1A). Nuclear IRF1 was present in 20% (ranging from 5% to 30%) of all uninfected macrophages, which is in line with previous results (6, 7). A significant increase was observed in infected macrophages over the time course and, surprisingly, also in the bystander population of uninfected macrophages (Fig. 1B, gray solid and patterned charts, respectively). Activation of uninfected bystander cells may result from soluble M. avium ligands acting on PRRs or factors secreted from infected cells, like inflammatory cytokines. In previous studies, we found that silencing of TLR7/8 or MyD88 (7) or inhibition of IKKβ (6) reduced IRF1 activation, suggesting that mycobacterial ligands can directly activate IRF1 through TLR signaling, as was also shown by others (16, 20, 34). However, IKKβ conveys signals from TLRs, IL-1R, and TNFR1 (19), and the pronounced activation in uninfected bystander cells prompted us to ask if activation could also be driven or enhanced through paracrine cytokine signaling. We thus assessed cytokine production during the time course of M. avium infection using a multiplex enzyme-linked immunosorbent assay (ELISA) approach. We found early secretion of TNF-α and IL-10, followed by strong and sustained secretion of IL-6 (Fig. 1C and see Fig. S1 in the supplemental material). The chemokines IL-8 (CXCL8), IP-10 (CXCL10), MCP-1, GRO-α (CXCL1), MIP-1α/β (CCL3/4), and SDF-1α (CXCL12) peaked 1 to 3 days postinfection (Fig. S1). IFN-β was secreted at low levels that did not vary significantly during infection, whereas IFN-α and IL-1α/β were produced in limited amounts and with kinetics similar to those of IL-6 and IP-10 (Fig. S1). Comparable results were found when cytokine gene expression was measured using real-time PCR, with no significant induction of type I IFNs but strong induction of *TNF* and *IL-6* (Fig. S2). We next treated macrophages with the recombinant (r) cytokine rIL-6, rTNF-α, rIFN-β, rIL-10, or rIP-10 and monitored IRF1 nuclear localization by confocal microscopy. Both rIL-6 and rTNF-α induced IRF1 activation to the same level as or to a level higher than that induced by rIFN-β (Fig. 1D). In contrast, neither rIP-10 nor rIL-10 induced IRF1 translocation to the nucleus (Fig. 1D).

10.1128/mBio.02121-21.1 FIG S1 Cytokine production by M. avium-infected macrophages. Human MDMs were infected with M. avium-DsRed for 10 min, followed by a chase for the indicated times. Supernatants were collected and subjected to multiplex ELISA. Bar charts represent average concentrations with standard errors of the means from 8 independent donors. Inflammatory cytokines are in black, chemokines in green, anti-inflammatory cytokines in blue, and interferons in red. *P* values between uninfected and different time points were calculated using the nonparametric ANOVA test. *, *P < *0.05; **, *P < *0.01. Download FIG S1, TIF file, 0.3 MB. Copyright © 2021 Gidon et al. 2021 Gidon et al. https://creativecommons.org/licenses/by/4.0/ This content is distributed under the terms of the Creative Commons Attribution 4.0 International license.

10.1128/mBio.02121-21.2 FIG S2 Gene expression of TNF-α, IL-6, and interferons in M. avium-infected macrophages. Human MDMs were infected with M. avium-DsRed for 10 min, followed by a chase for the indicated times. Total RNAs were collected, and GAPDH, IFN-α/β/γ (A) and GAPDH, TNF-α, and IL-6 (B) mRNA production was assessed by real-time PCR. Dots represent CT values, and bar charts represent averages of results from at least 4 individual donors. *P* values between noninfected cells at different time points were calculated using the nonparametric paired ANOVA test. *, *P* < 0.05; **, *P* < 0.01; ***, *P* < 0.001. Download FIG S2, TIF file, 0.2 MB. Copyright © 2021 Gidon et al. 2021 Gidon et al. https://creativecommons.org/licenses/by/4.0/ This content is distributed under the terms of the Creative Commons Attribution 4.0 International license.

**FIG 2:**
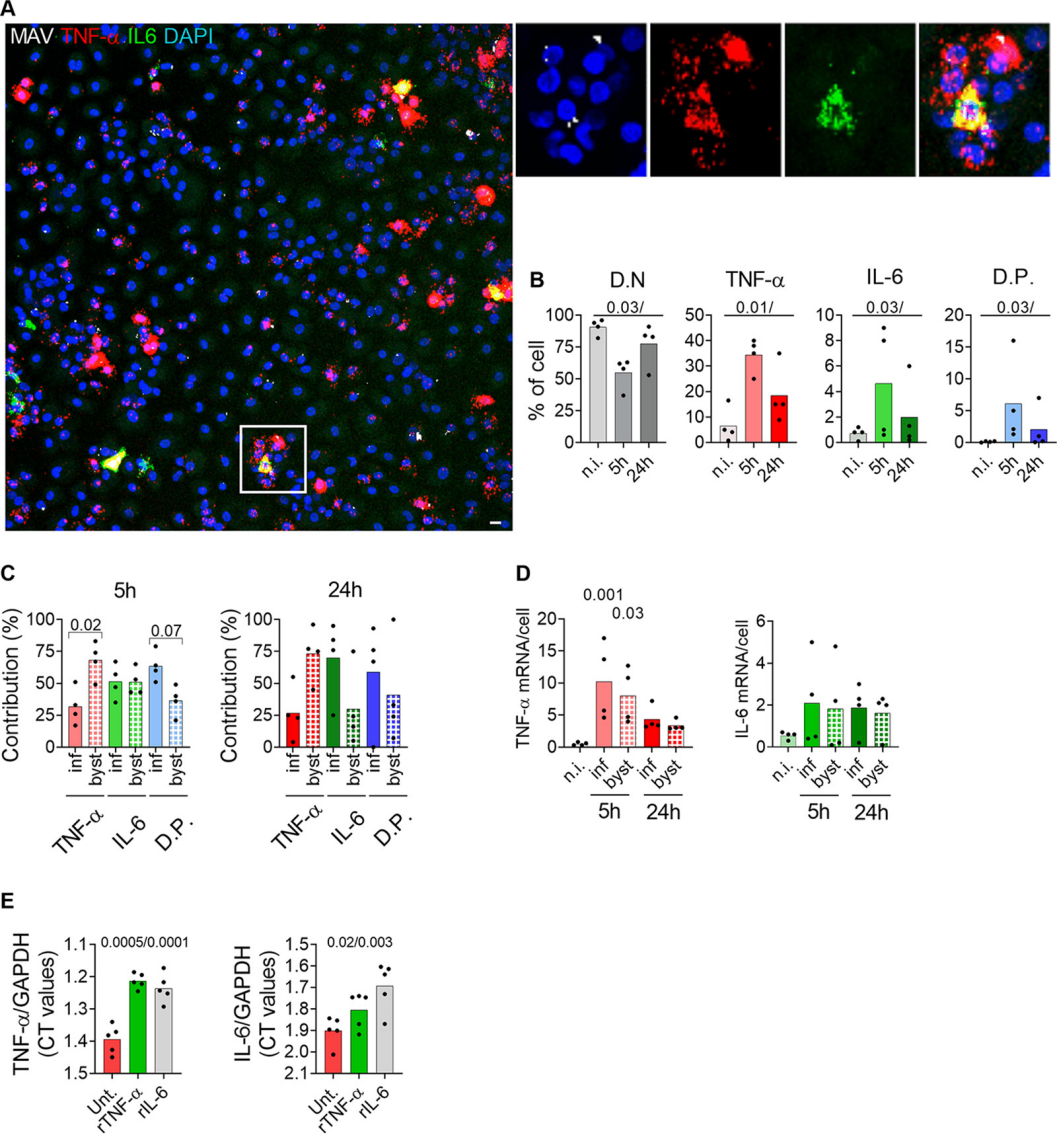
Infected and uninfected bystander macrophages contribute to IL-6 and TNF-α production. Human MDMs were infected with M. avium-CFP for 10 min, followed by a chase for 5 h and 24 h. TNF-α and IL-6 mRNA were revealed using RNAscope. (A) Merged images showing nuclei (blue), M. avium-CFP (white), TNF-α (red), and IL-6 (green). The magnified insets show single and merged channels. (B) Quantifications of double-negative (D.N; gray), TNF-α-positive (red), IL-6-positive (green), and TNF-α/IL-6 double-positive (D.P.; blue) macrophages as percentages of the total population. Light, intermediate, and dark shades represent noninfected cells (n.i.) and cells 5 h and 24 h postinfection, respectively. (C) Relative contributions of infected and uninfected bystander cells from positive populations (TNF-α, red; IL-6, green; DP, blue) at 5 h and 24 h postinfection. (B and C) Shaded bars represent infected cells, and patterned bars represent uninfected bystander cells. Dots represent average cell counts per individual donor (the total cell number was at least 500 per donor and per time point), and bar charts represent averages from 4 individual donors. (D) Quantification of the number of TNF-α and IL-6 mRNA molecules per macrophage for the different time points. Dots represent average mRNA counts per individual donor (the total cell number was at least 500 per donor and per time point), and bar charts represent averages from 4 individual donors. (E) Effect of rTNF-α and rIL-6 on TNF-α and IL-6 production. Human MDMs were treated with 25 nM rTNF-α (red) or rIL-6 (green) for 4 h. Levels of TNF-α and IL-6 expression were probed by real-time PCR. Bar charts represent CT values for TNF-α and IL-6 normalized to that for GAPDH (*n* = 5 donors). *P* values were calculated using the paired nonparametric ANOVA test. The scale bar represents 10 μm.

**FIG 3:**
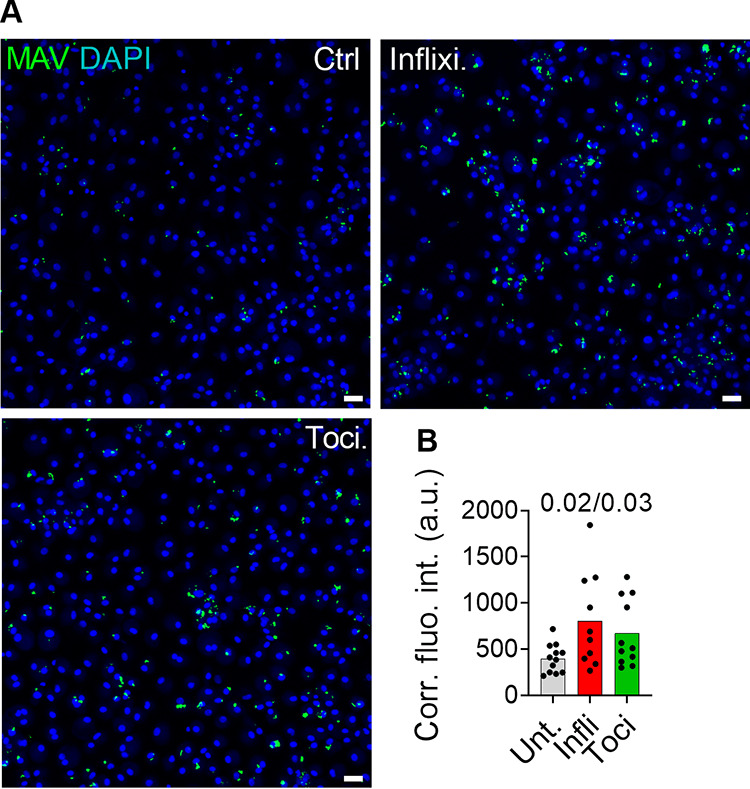
Inhibition of TNF-α or IL-6 increases M. avium intracellular growth. Human MDMs were infected with live M. avium-DsRed for 10 min, followed by a chase of up to 72 h in the presence of 2 μg/ml infliximab or tocilizumab. M. avium intracellular growth was monitored by confocal microscopy. (A) Merged images showing Hoechst stain (blue) and M. avium (green). (B) Quantification of mycobacterial loads. Dots represent the average corrected fluorescence intensity (Corr. fluo. int.) (in arbitrary units [a.u.]) per individual donor (>500 cells per donor and per time point), and bar charts represent the average of 10 individual donors who were untreated (gray) or administered infliximab (red) or tocilizumab (green). *P* values between untreated and treated conditions were calculated using the nonparametric paired ANOVA test. Scale bars represent 10 μm.

To test if TNF-α or IL-6 was involved in the activation of IRF1, we first treated M. avium-infected macrophage cultures with infliximab, a humanized antibody that neutralizes TNF-α. Compared to what occurred with untreated cells, infliximab significantly reduced IRF1 activation in bystander cells and infected cells 4 h to 48 h postinfection (Fig. 1E). In fact, considering that nuclear IRF1 is present in 5 to 30% of noninfected macrophages (Fig. 1E), inhibition by infliximab was even more pronounced. However, infliximab even diminished constitutive nuclear IRF1 in resting macrophages, making it difficult to accurately quantify to what extent infliximab completely inhibited M. avium-induced IRF1 activation. Similar results were obtained with tocilizumab, a humanized mouse antibody that blocks the IL-6 receptor (IL-6R) (Fig. 1F), and a Janus kinase inhibitor (JAK I) (Fig. S3). Overall, a more pronounced inhibition of IRF1 activation was obtained by combined treatment with tocilizumab and infliximab (Fig. 1G), especially in bystander cells. Taken together, these data suggest that IL-6 and TNF-α are required for full IRF1 activation in M. avium-infected macrophages and for driving activation in uninfected bystander cells. To identify the cellular source of cytokine production, we used multiplex fluorescence mRNA hybridization against IL-6 and TNF-α (green and red, respectively, in Fig. 2). By counting fluorescent dots, which represent mRNA molecules, we observed an increase after 5 h in cells transcribing either TNF-α (35%), IL-6 (5%), or both cytokines (6%, double producers) (red, green, and blue bars, respectively, in Fig. 2B) and a decrease 24 h postinfection. Both the infected cells and bystander cells contributed to cytokine production, but whereas TNF-α mRNA was more frequent in bystander cells at both time points, a larger fraction of infected cells expressed IL-6 at 24 h postinfection (Fig. 2C). Quantification of the number of mRNA molecules per cell did not reveal any significant differences between infected and bystander cells (Fig. 2D). Finally, we found that rTNF-α and rIL-6 could equally well drive TNF expression, whereas rIL-6 was more efficient in driving IL-6 expression (Fig. 2E). Seen together with cytokine secretion profiles (Fig. S1), these results suggest that TNF-α from uninfected bystander cells is an early driving force of this autocrine/paracrine signaling loop, which is later sustained/dominated by IL-6 produced mainly by infected cells. To investigate if TNF-α and IL-6 signaling contributes to controlling the infection, we treated infected macrophages with infliximab or tocilizumab and measured the fluorescence intensity of M. avium-DsRed to assess the burden per infected cell (Fig. 3A). Image quantification showed a significant increase of intracellular M. avium in cells treated with infliximab (108%, *P = *0.02) or tocilizumab (74%, *P = *0.03) compared to levels in untreated cells (Fig. 3B). Taken together, our data show that TNF-α and IL-6 are produced by infected and bystander macrophages and are needed to control M. avium infection, possibly in part by controlling IRF1 activation.

10.1128/mBio.02121-21.3 FIG S3 The JAK inhibitor reduces IRF1 activation. Human MDMs were infected with live M. avium-DsRed for 10 min, followed by a chase for up to 48 h in the presence of 15 nM JAK I inhibitor (green). The nuclear translocation of IRF1 was analyzed by confocal microscopy at the indicated time points. Dots represent the average value per donor (>500 cells per time point), and bar charts represent averages of results for 5 different donors. *P* values between noninfected cells at different time points were calculated using the nonparametric ANOVA test. Download FIG S3, TIF file, 1.4 MB. Copyright © 2021 Gidon et al. 2021 Gidon et al. https://creativecommons.org/licenses/by/4.0/ This content is distributed under the terms of the Creative Commons Attribution 4.0 International license.

### IRF1 is involved in controlling M. avium infection via the induction of IRG1.

**FIG 4:**
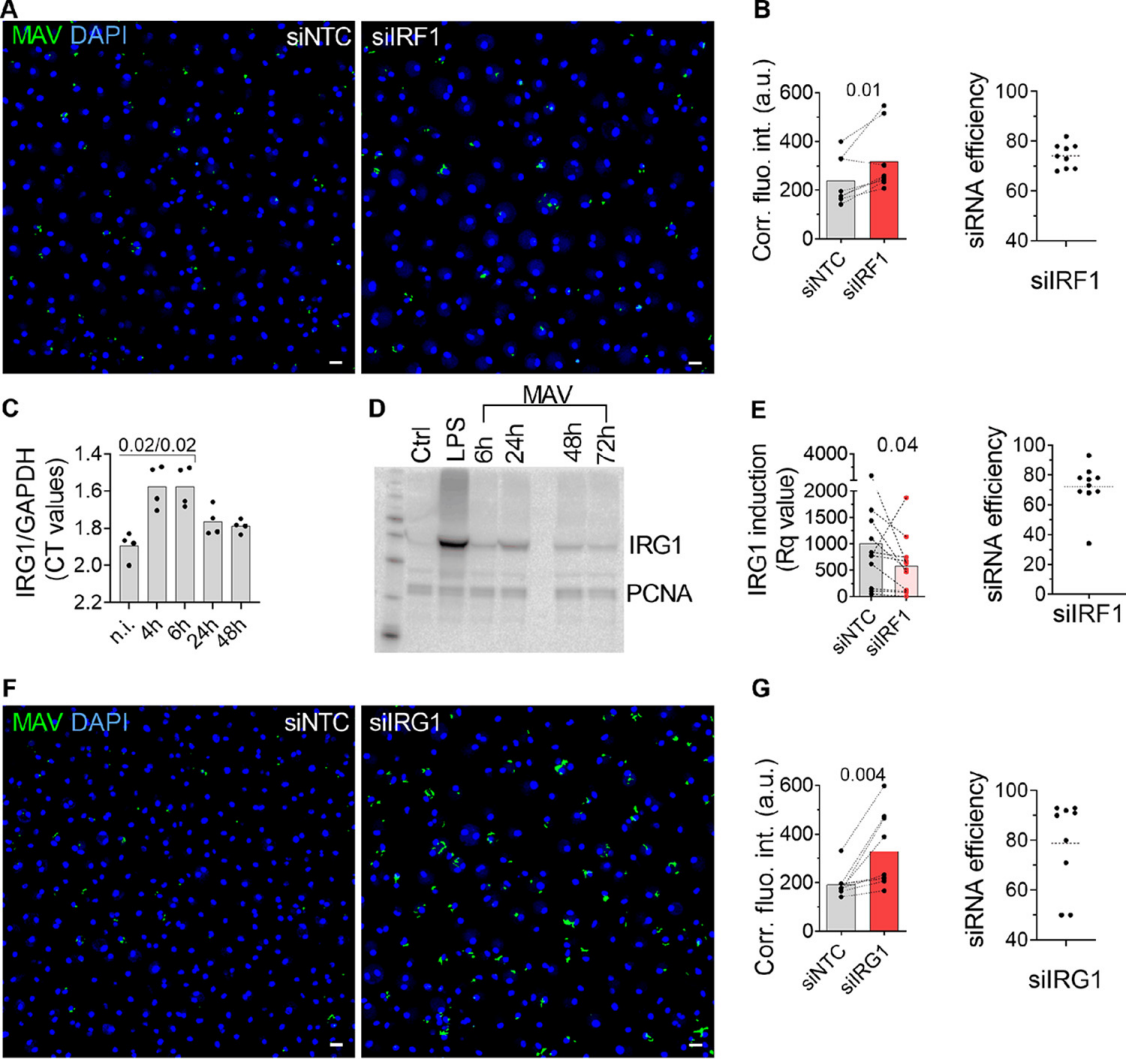
IRF1 contributes to controlling M. avium infection. (A, B) Human MDMs pretreated with siRNA against *IRF1* were infected with M. avium-DsRed for 10 min, followed by a chase of 72 h. M. avium intracellular growth was monitored by confocal microscopy. (A) Merged images showing Hoechst stain (blue) and M. avium (green). (B) Quantification of mycobacterial loads. Dots represent the average corrected fluorescence intensity (arbitrary unit) per individual donor (>500 cells per donor and per time point), and bar charts represent averages of results for 9 individual donors for the nontargeting control siRNA (siNTC, gray) and siIRF1 (red). siRNA efficiency for each individual donor was tested by real-time PCR and plotted as a percentage decrease from the level for the siNTC (B, right). The *P* value between the siNTC and siIRF1 conditions was calculated using the nonparametric Wilcoxon paired signed-rank test. (C) *IRG1* mRNA expression in human MDMs 4 h, 6 h, 24 h, 48 h, and 72 h postinfection was assessed by real-time PCR. Bar charts represent averages of IRG1 CT values normalized to that for GAPDH of 4 donors per time point. IRG1 was not detected in uninfected control cells and set arbitrarily at 40 CT to point out the increase in IRG1 (D). Western blot analysis shows IRG1 protein expression 4 h, 6 h, 24 h, 48 h, and 72 h postinfection. Uninfected human MDMs were challenged with 100 ng/ml LPS for 24 h as a positive control for IRG1 production. The blot is representative of 2 independent experiments. (E) Human MDMs pretreated with siNTC or against IRF1 (siIRF1) were infected with M. avium-DsRed for 10 min, followed by a chase of 4 h. *IRG1* expression were assessed by real-time PCR. Dots represent *Rq* values for IRG1 induction. Bar charts represent the averages of results from 11 individual donors for IRG1 (red). (F, G) Human MDMs pretreated with siRNA against IRG1 were infected with M. avium-DsRed for 10 min, followed by a chase of 72 h. Intracellular growth was monitored by confocal microscopy. (F) Merged images showing Hoechst stain (blue) and M. avium (green). (G) Dots represent the average corrected fluorescence intensity (in arbitrary units) per individual donor (>500 cells per donor and per time point), bar charts represent the averages of results for 9 individual donors for siNTC (gray) and siIRG1 (red). siRNA efficiency for each individual donor was tested by real-time PCR and plotted as a percentage of decrease from that for siNTC (G, right). *P* values between siNTC and siIRG1 conditions were calculated using the nonparametric Wilcoxon paired signed-rank test. *, *P < *0.05; **, *P < *0.01. Scale bars represent 10 μm.

We next tested if IRF1 activation is involved in controlling M. avium infection by treating macrophages with small interfering RNA (siRNA) against IRF1 prior to infection with M. avium-DsRed for 3 days. We measured the fluorescence intensity of M. avium-DsRed to assess the burden per infected cell (Fig. 4A). Image quantification showed a significant increase of intracellular M. avium (45%, *P = *0.001) in cells where IRF1 was silenced (75% reduction) compared to levels in cells treated with the nontargeting control (Fig. 4B).

IRF1 is reported to be a mediator of interferon responses (15–20, 35–38). However, the clinical M. avium strain used in our studies, M. avium 104, is a poor inducer of IFNs in human primary macrophages, suggesting that alternative functions of IRF1 may be important for antimycobacterial defenses. Michelucci and colleagues have postulated that the mitochondrial enzyme IRG1, which produces the putatively antimicrobial metabolite itaconate (23), is under the control of IRF1 (22). Here, we first found that *IRG1* mRNA is induced in M. avium-infected human macrophages at 4 h and 6 h (average threshold cycle [CT] values of 31 and 32, respectively) and decreased after 24 h of infection (Fig. 4C). Western blot analysis confirmed the production of IRG1 protein after 6 h, with a peak at 24 h postinfection (Fig. 4D). Furthermore, knockdown of *IRF1* (70% reduction) prior to M. avium infection led to a 45% decrease in *IRG1* expression (Fig. 4E), confirming that IRF1 is involved in driving IRG1 expression.

To investigate the role of IRG1 during M. avium infection, we next treated macrophages with siIRG1 prior to M. avium-DsRed infection for 3 days. After fixation, we measured the fluorescence from M. avium-DsRed to assess the burden per infected macrophage (Fig. 4F). Image quantification showed a significant increase (70%, *P = *0.005) in the M. avium burden in IRG1-depleted cells (80% reduction) compared to that in cells treated with a nontargeting control (Fig. 4G). The effect was even more pronounced in bone marrow-derived macrophages (BMDMs) from IRG1 knockout mice; 3 days postinfection, IRG1-deficient macrophages showed a 140%-increased mycobacterial load compared to that of wild-type cells (Fig. S4). Collectively, these data show that IRF1 activity is involved in IRG1 expression and that both are involved in controlling M. avium infection in human primary macrophages.

10.1128/mBio.02121-21.4 FIG S4 IRG1 contributes to control the intracellular growth of M. avium in mouse macrophages. Mouse BMDMs from a wild-type or *Irg1* knockout background were infected with M. avium-DsRed for 10 min, followed by a chase of 72 h. Intracellular growth was monitored by confocal microscopy. Dots represent the average cell count per individual mouse (*n* > 500), and bar charts represent averages of results for 3 control mice (grey) and IRG1 knockout mice (red). The *P* value between wild-type and IRG1 knockout mice was calculated using the parametric *t* test. **, *P* < 0.01. Download FIG S4, TIF file, 1.2 MB. Copyright © 2021 Gidon et al. 2021 Gidon et al. https://creativecommons.org/licenses/by/4.0/ This content is distributed under the terms of the Creative Commons Attribution 4.0 International license.

### IRG1 expression is induced by TLR activation and TNF-α and IL-6 autocrine/paracrine signaling.

**FIG 5:**
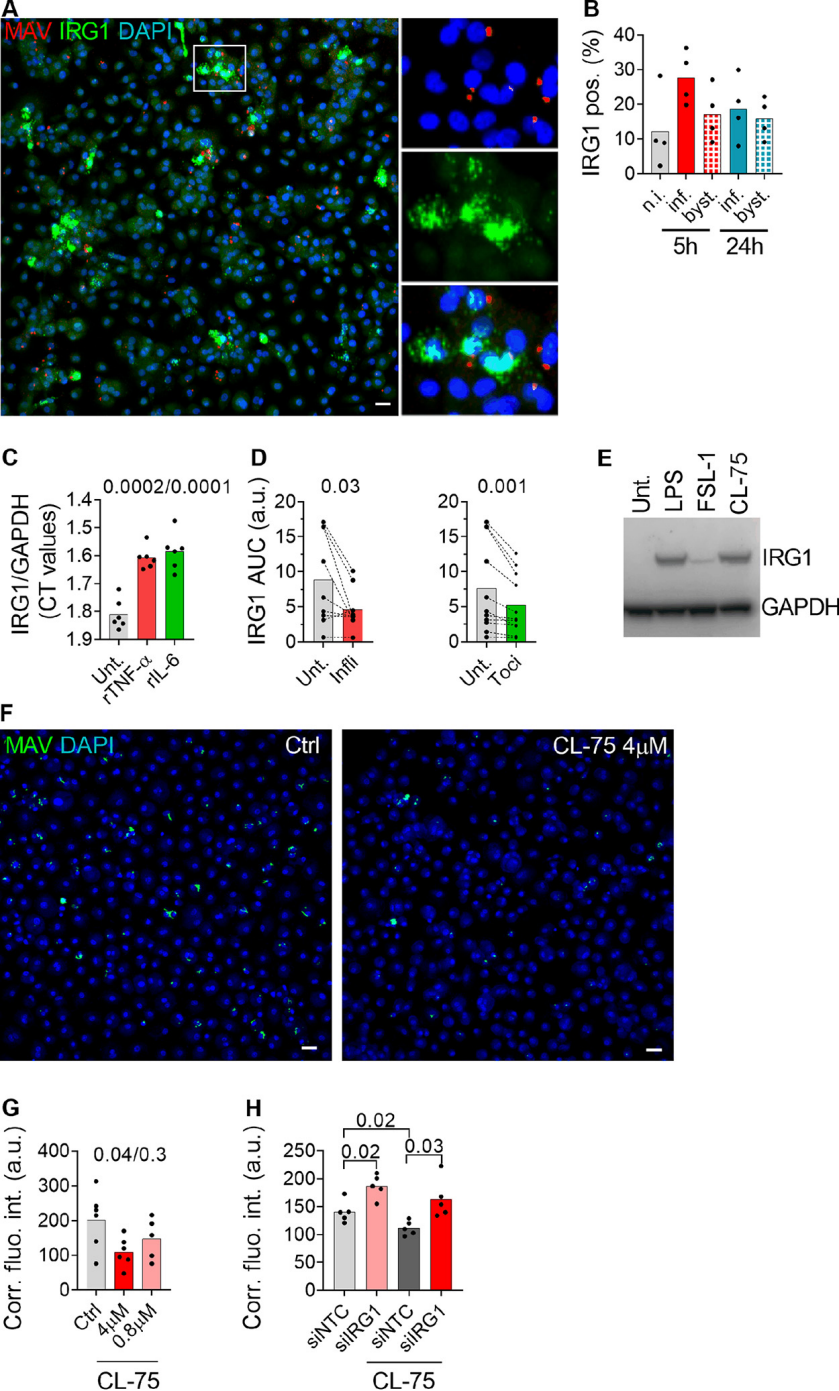
TNF-α and IL-6 contribute to IRG1 induction. Human MDMs were infected with M. avium-CFP for 10 min, followed by chase of 5 h and 24 h. IRG1 mRNA was revealed using RNAscope. (A) Merged images showing nuclei (blue), M. avium-CFP (red), and IRG1 (green). The magnified panels of the inset show single and merged channels. (B) Contributions of the infected and uninfected bystander populations at 5 h and 24 h postinfection. Filled bars represent infected cells, and patterned bars represent bystander cells. Dots represent the average cell count per individual donor (the total cell number was at least 500 per donor and per time point), and bar charts represent averages of results for 4 individual donors. pos., positive. (C) Human MDMs were treated with 25 nM rTNF-α or rIL-6 (red or green, respectively) for 4 h. IRG1 expression was probed by real-time PCR. Bar charts represent average IRG1 CT values normalized to the value for GAPDH for rTNF-α and rIL-6 from 6 independent donors. (D) Human MDMs were infected with M. avium-CFP for 10 min, followed by a chase of 4 h, 6 h, or 24 h and cotreated with infliximab or tocilizumab (red and green, respectively). IRG1 expression was probed by real-time PCR and quantified by calculating the area under the curve of relative expression values (arbitrary units). Bar charts represent average area under the curve values for infliximab and tocilizumab from 7 independent donors (red and green, respectively). *P* values between the control and infliximab/tocilizumab groups were calculated using the nonparametric Wilcoxon paired signed-rank test. (E) Human MDMs were challenged with 100 ng/ml LPS, 50 ng/ml FSL-1, or 8 μM CL-75 for 24 h, and IRG1 expression was probed by Western blotting. The blot is representative of 3 independent experiments. (F) Human MDMs were infected with live M. avium-DsRed for 10 min, followed by a chase of up to 72 h, and cotreated with 4 μM or 0.8 μM CL75. M. avium intracellular growth was monitored by confocal microscopy. (F) Merged images showing Hoechst stain (blue) and M. avium (green). (G) Dots represent the average corrected fluorescence intensity (arbitrary units) per individual donor (>500 cells per donor and per time point), and bar charts represent averages of results for 6 individual donors who were untreated (gray) or treated with 4 μM (red) and 0.8 μM (light red) CL-75. (H) Human MDMs pretreated with siRNA against IRG1 were infected with M. avium-DsRed for 10 min, followed by a chase of 72 h, and cotreated with 4 μM CL-75. Intracellular growth was monitored by confocal microscopy. Dots represent the average cell count per individual donor (>500 per donor and per time point), and bar charts represent averages of results for cells from 9 individual donors that were not treated (gray) or treated with siIRG1 (light red), siNTC plus 4 μM CL-75 (dark gray), and siIRG1 4 μM CL-75 (dark red). *P* values between untreated and treated conditions were calculated using the nonparametric paired ANOVA test. Scale bars represent 10 μm.

To obtain full activation of IRF1 in M. avium-infected and bystander macrophages, TNF-α and IL-6 signaling pathways are needed (Fig. 1E to H). Further, since IRF1 seems to be central in driving IRG1 expression, we hypothesized that TNF-α and IL-6 autocrine/paracrine signaling would also be involved. We first assessed the cellular source of *IRG1* expression using fluorescence *in situ* hybridization (Fig. 5A). Quantification shows that about 30% of the infected macrophages (Fig. 4B, dark red) and 20% of the bystander macrophages (Fig. 5B, light red) expressed IRG1 mRNA at 5 h postinfection. Next, we treated uninfected macrophage cultures with 25 nM rTNF-α or rIL-6 and found that both cytokines could independently induce *IRG1* expression (average CT values of 34 and 32, respectively) (Fig. 5C). We then treated M. avium-infected macrophages with infliximab or tocilizumab and assessed *IRG1* expression 4, 6, and 24 h postinfection. Blocking IL-6R or TNF-α significantly reduced *IRG1* expression, with an average effect of 32% in both cases (Fig. 5D, area under the curve of relative quantity [*Rq*] values).

As for IRF1, a more direct activation of *IRG1* expression through PRR engagement by M. avium is plausible. We and others have shown that TLR2 and TLR8 are central in sensing and controlling M. avium infection (4, 7, 39). Treatment of uninfected macrophages with the TLR2 ligand FSL-1 or the TLR8-ligand CL-75 induced IRG1 protein production (Fig. 5E). However, the magnitudes of activation were different, with CL-75 inducing a response equivalent to that of lipopolysaccharide (LPS) (TLR4 ligand) and with FSL-1 only weakly increasing IRG1. Taken together, these results suggest that during M. avium infection, TLR engagement initially activates IRF1/IRG1 along with the secretion of TNF-α and IL-6. TNF-α and IL-6 subsequently activate a feed-forward signaling loop that reinforces the expression of IRF1/IRG1 in infected and bystander macrophages. Given that IRG1 is needed to control the infection and that CL-75 is a strong IRG1 inducer, we hypothesized that strengthening the activation of TLR8 beyond that of M. avium itself may improve the macrophage control of infection. Activation of TLR8 with 4 μM CL-75 induced a 50% reduction of the intracellular M. avium burden after 3 days of infection (Fig. 5F and G). The antimicrobial effect of CL-75 was reduced in macrophages depleted of IRG1 using siRNA, suggesting that IRG1 activity contributed to the growth impairment (Fig. 5H).

### Itaconate supplement inhibits M. avium growth in culture but not in macrophages.

**FIG 6:**
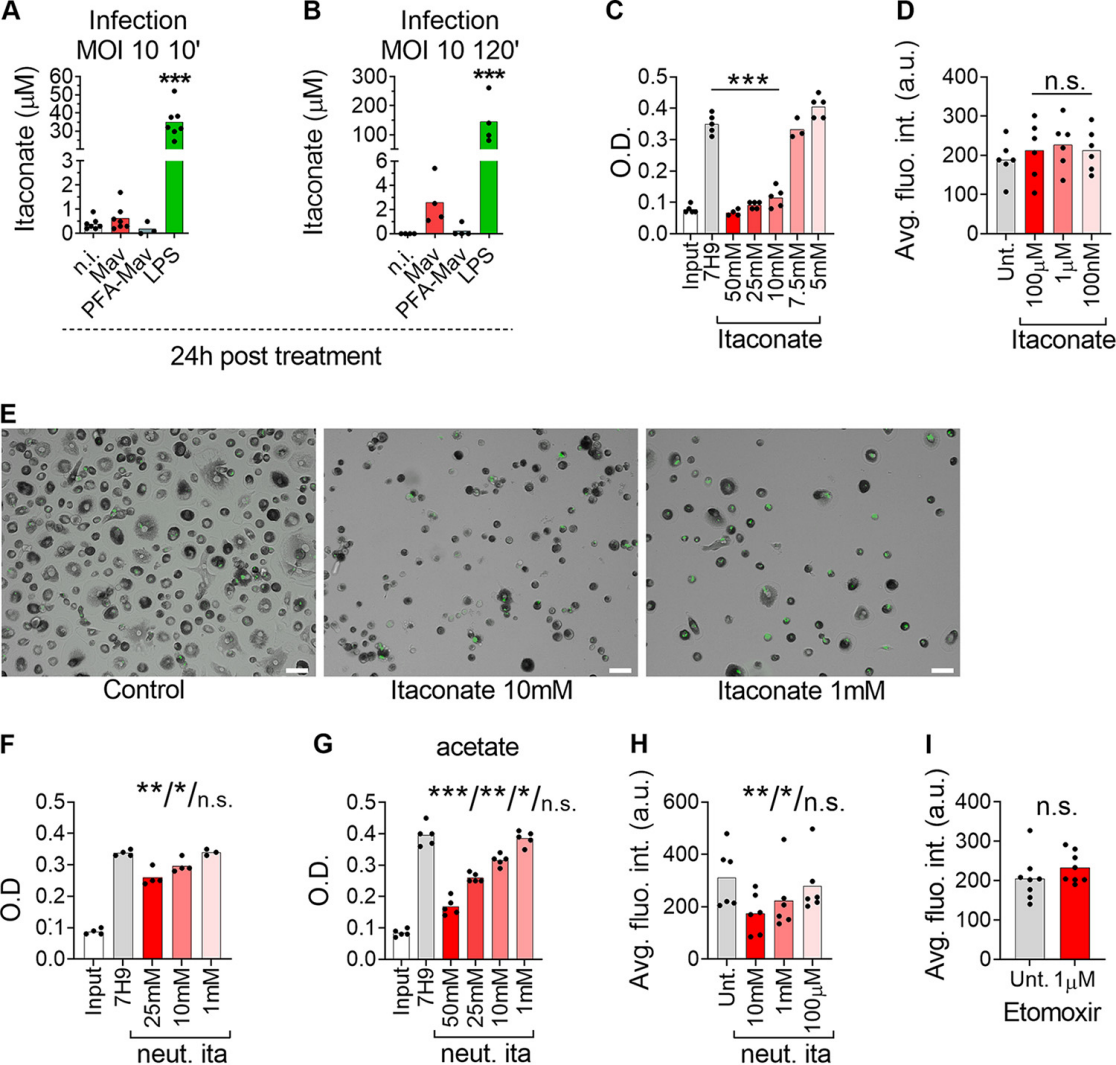
Antimycobacterial activity of exogenously added itaconate. (A, B) Human MDMs were challenged with 100 ng/ml LPS (green bars) or infected with M. avium-DsRed (red bars) or PFA-killed M. avium-DsRed (blue bars) for 10 min (A) or 120 min (B), followed by a chase of 24 h. Bar charts show intracellular itaconate levels (μM) measured in cell extracts 24 h postchallenge from at least 4 individual donors using capillary ion chromatography (A) and liquid chromatography (B) coupled to tandem mass spectrometry. (C) M. avium liquid cultures were treated with the indicated concentrations of itaconate. Bars charts show optical density measurements (O.D.) at 24 h from 5 independent experiments. (D) Human MDMs treated with itaconate were infected with M. avium-DsRed for 10 min, followed by a chase of 72 h. Intracellular growth was monitored by confocal microscopy. Dots represent the average fluorescence intensity per individual donor (>500 cells per donor and per time point), and bar charts represent the average of at least 6 individual donors. The *P* value between untreated and treated conditions was calculated using the nonparametric Wilcoxon paired signed-rank test. (E) Human MDMs treated with 10 or 1 mM itaconate were infected with M. avium-DsRed for 10 min, followed by a chase of 72 h. (F, G) M. avium liquid cultures with glycerol (F) or acetate (G) as the carbon source treated with the indicated concentrations of pH-neutralized itaconate (neut. ita). Bars charts show optical density measurements at 24 h from 4 independent experiments. (H) Human MDMs were infected with live M. avium-DsRed for 10 min and cotreated with 10 mM, 1 mM, and 100 μM neutralized itaconate. M. avium intracellular growth was monitored by confocal microscopy. Dots represent the average corrected fluorescence intensity (arbitrary units) per individual donor (>500 cells per donor and per time point), and bar charts represent the averages of results from 6 individual donors whose cells were untreated (gray) or treated with neutralized itaconate (red). (I) Human MDMs were infected with live M. avium-DsRed and cotreated with 1 μM etomoxir. M. avium intracellular growth was monitored by confocal microscopy. Dots represent the average corrected fluorescence intensity (arbitrary units) per individual donor (>500 cells per donor and per time point), and bar charts represent the average of results for 8 individual donors whose cells were untreated (gray) and treated with etomoxir (red). *P* values between untreated and treated conditions were calculated using the nonparametric Wilcoxon paired signed-rank test. *, *P < *0.05; **, *P < *0.01. The scale bar represents 10 μm. neut.

In their seminal paper, Michelucci and colleagues demonstrated that *Irg1* encodes the enzyme IRG1, which is responsible for the production of itaconate, a metabolite with potential antimicrobial and immunomodulatory properties (23–25, 27, 30, 32, 40, 41). We therefore assessed the endogenous accumulation of itaconate in human MDMs during M. avium infection by tandem mass spectrometry. We applied two different chromatographic separation techniques prior to mass spectrometric detection in all samples to validate concentrations close to the noise level (Fig. S5). LPS is reported to be a strong inducer of itaconate and was included as a positive control. Accordingly, 4-h and 24-h activation with 100 ng/ml LPS induced high endogenous concentrations of itaconate (Fig. 6A, green bar; Fig. S6A). Our regular infection protocol yields 20 to 40% infected cells, which is ideal for comparing infected and bystander cell populations. However, using our standard protocol of infection (multiplicity of infection [MOI] of 10 for 10 min), we measured low levels of itaconate in macrophages both 4 h and 24 h after M. avium infection, which did not significantly differ from the levels in uninfected macrophages (Fig. 6A, red bar; Fig. S6A). To test if the infectivity, despite the induction of IRG1, was too low to induce elevated levels of itaconate, we adjusted our protocol to obtain virtually all macrophages infected (MOI of 10 for 2 h). Using this condition, we observed some accumulation of itaconate 24 h after M. avium infection (∼3 μM) (Fig. 6B, red bar), although it was still 50 times lower than the accumulation induced by LPS (∼150 μM) (Fig. 6B, green bar). To test if the low levels could be due to consumption or degradation of itaconate by M. avium, macrophages were also challenged with paraformaldehyde (PFA)-killed bacteria, after we verified that PFA-killed M. avium induces *IRG1* expression (Fig. S6B). Infection with PFA-killed M. avium showed less itaconate accumulation than live M. avium, discarding the possibility of itaconate consumption or degradation by the mycobacteria (Fig. 6A and B, blue bars).

10.1128/mBio.02121-21.5 FIG S5 Representative capillary ion chromatography-tandem mass spectrometry (capIC-MS/MS) and liquid chromatography tandem mass spectrometry (LC-MS/MS) itaconate chromatograms. Human MDMs were challenged with 100 ng/ml LPS and infected with live or PFA-killed M. avium-DsRed for 10 min or 120 min, followed by a chase of 24 h. Itaconate concentrations in all cell extracts, collected 24 h postchallenge, were quantified both by capIC-MS/MS and LC-MS/MS. The chromatograms of the quantifier transition from 129 to 85, normalized to the largest of the peaks, is shown for a representative cell extract from all treatment groups and the analytical grade standard at a concentration of 5 μM. Traces are representative of at least 4 independent experiments. Download FIG S5, TIF file, 2.9 MB. Copyright © 2021 Gidon et al. 2021 Gidon et al. https://creativecommons.org/licenses/by/4.0/ This content is distributed under the terms of the Creative Commons Attribution 4.0 International license.

10.1128/mBio.02121-21.6 FIG S6 PFA-killed M. avium induces IRG1. (A) Human MDMs were challenged with 100 ng/ml LPS (green bars) or infected with live M. avium-DsRed (red bars) or PFA-killed M. avium-DsRed (blue bars) for 10 min, followed by a chase of 4 h. Bar charts show intracellular itaconate (micromolar) levels measured in cell extracts 4 h postchallenge from at least 4 individual donors using capillary ion chromatography-tandem mass spectrometry. (B) Human MDMs were infected with live or PFA-killed M. avium-DsRed for 10 min, followed by a chase of 4 h and 24 h. *IRG1* mRNA expression levels were assessed by real-time PCR. Dots represent CT values, and bar charts represent averages of results for 4 individual donors. (C) M. avium liquid cultures were treated with the indicated concentrations of 4-octyl itaconate (4-OI) for 24 h. Growth measured as the optical density (OD) from 5 independent experiments is shown. (D) Human MDMs treated with the indicated concentrations of itaconate were infected with M. avium-DsRed for 10 min, followed by a chase of 72 h. Intracellular growth was monitored by CFU plating. (E) Human MDMs treated with various concentrations of 4-OI were infected with M. avium-DsRed for 10 min, followed by a chase of 72 h. M. avium intracellular growth was monitored by confocal microscopy. Dots represent the average corrected fluorescence intensity (arbitrary units) per individual donor (>500 cells per donor and per time point), and bar charts represent averages of results from 7 individual donors who were untreated (gray) and treated with 4-OI (red). *P* values between the untreated and treated conditions were calculated using the parametric (A to D) and nonparametric (E) paired ANOVA test. *, *P* < 0.05; **, *P* < 0.01. Download FIG S6, TIF file, 2.4 MB. Copyright © 2021 Gidon et al. 2021 Gidon et al. https://creativecommons.org/licenses/by/4.0/ This content is distributed under the terms of the Creative Commons Attribution 4.0 International license.

Itaconate is shown to have bacteriostatic activity toward M. tuberculosis in liquid culture under conditions requiring metabolic reprogramming via the glyoxylate shunt pathway, the methyl citrate cycle, and/or cholesterol catabolism (23, 41), although a direct antimycobacterial role of itaconate has been questioned by Nair et al. (33). We therefore tested if itaconate had growth-inhibiting effects on M. avium by providing it exogenously. In line with the findings of Michelucci et al. (23) and Ruetz et al. (41), we show that itaconate inhibits M. avium growth in liquid cultures in a dose-dependent manner, with a complete inhibition at 10 to 50 mM itaconate (Fig. 6C). Growth inhibition was achieved in regular culture medium (Middlebrook 7H9 plus albumin dextrose catalase [ADC]) without a change of the carbon source. We also tested the membrane-permeable 4-octyl-itaconate (4-OI) derivative (27). In liquid M. avium cultures, we observed a dose-dependent inhibition of growth that was complete at 750 μM 4-OI (Fig. S6C). We then treated macrophages with 100 nM, 1 μM, or 100 μM itaconate or 4-OI during infection with M. avium*-*DsRed and measured the bacterial loads after 3 days. To our surprise, quantification of bacterial load by fluorescence or CFU counting did not reveal any effect of itaconate or 4-OI on intracellular M. avium growth (Fig. 6D; Fig. S6D and E). High (millimolar) concentrations of itaconate were needed for inhibition of M. avium in culture, and similar concentrations were toxic to macrophages, most likely due to acidification of the culture medium (Fig. 6E). It was recently shown that millimolar concentrations of itaconate acidify the medium but also that the bactericidal activity of itaconate is enhanced under acidic conditions, such as in the phagolysosome (42, 43). In line with this, the neutralization of itaconate using NaOH reduced the growth-inhibitory effect of M. avium in liquid culture (Fig. 6F; compare this panel to Fig. 6C). However, neutralized itaconate showed a strong inhibition of M. avium replication when acetate was used as a carbon source to force the bacteria to use the glyoxylate shunt (Fig. 6G). Higher concentrations of neutralized itaconate were also well tolerated by the macrophages and inhibited the intracellular growth of M. avium at 1 to 10 mM, similar to what was achieved in culture (Fig. 6 h). Taken together, we confirm that high concentrations of itaconate inhibit M. avium growth both in culture and inside macrophages, most likely in a pH-dependent manner. However, based on the low levels accumulated endogenously in M. avium-infected macrophages, we cannot conclude that the antimycobacterial activity of IRG1 is mediated only by itaconate. Hall et al. have proposed an alternative function of IRG1 in regulating beta-oxidation-dependent mitochondrial reactive oxygen species (ROS) production, resulting in bactericidal activity toward intraphagosomal Salmonella (24). To explore this hypothesis, macrophages infected with M. avium were treated with a 1 μM concentration of a carnitine palmitoyltransferase 1 (CPT1) inhibitor, etomoxir (44). Quantification did not reveal a significant increase of the intracellular burden compared to that in the untreated condition (Fig. 6I), excluding a prominent role of beta-oxidation in the control of the infection.

**FIG 7:**
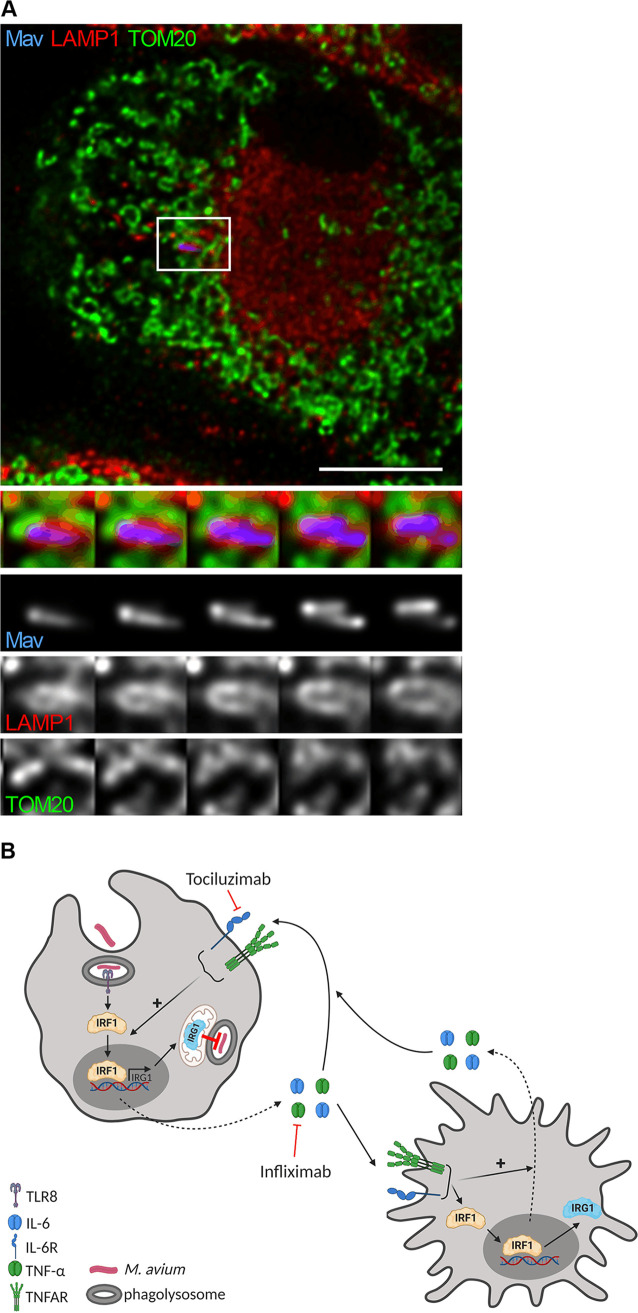
Human MDMs were infected with M. avium-CFP (blue) for 10 min, followed by a chase of 24 h. (A) LAMP1 (lysosomes, red) and TOMM20 (mitochondria, green) were stained using antibodies and analyzed by confocal microscopy. A merged image is shown. Bottom-to-top projections of 3D stacks from the boxed area are shown in the lower panels and represent the merged channel, M. avium, LAMP1, and TOMM20. The image is representative of 2 independent experiments. The scale bar represents 10 μm. (B) Working model. TNFAR, TNF-α receptor. This summary was created with BioRender.com.

Interestingly, Chen et al. recently expressed an itaconate biosensor in Salmonella and showed that itaconate was efficiently delivered to the Salmonella-containing vacuole (SCV) through intimate contact between IRG1-containing mitochondria and the SCV (25). Our attempts to stain for IRG1 in M. avium-infected macrophages failed, as the commercially available antibodies were not suitable for immunofluorescence. However, in line with the findings of Chen et al., Hall et al., and others (24, 25, 45), we observed repositioning of mitochondria to M. avium phagosomes (Fig. 7A), suggesting that directed delivery of IRG1-driven itaconate is plausible (Fig. 7B).

Taken together, our results suggest that an autocrine/paracrine loop mediated by TNF-α and IL-6 fuels the IRF1-IRG1-itaconate axis in M. avium-infected human macrophages, which contributes to controlling the mycobacterial load.

## DISCUSSION

Mycobacterial infections are heterogeneous, and only a fraction of the cells in a tissue or in a cell culture are usually infected. Still, we know that uninfected bystander cells respond to the infection through sensing of signals secreted by the infected cells, soluble microbial ligands, and/or humoral factors activated by the infection. The nature and impact of such cell-cell communication have been less investigated and require either trans-well setups or single-cell analyses of infected cell cultures or tissue. By applying confocal microscopy to study single-cell and subcellular events in M. avium-infected macrophages, we have identified a feed-forward signaling loop whereby TNF-α and IL-6 activate an antimycobacterial program driven by IRF1/IRG1 in infected and uninfected bystander macrophages.

Infliximab and tocilizumab, humanized antibodies that neutralize TNF-α and IL-6R, respectively, inhibited IRF1/IRG1 activation and increased the intracellular M. avium burden. This effect may partially explain the susceptibility of patients treated with anti-inflammatory drugs to mycobacterial infection (1, 2). TNF-α and IL-6 play key roles in mycobacterial host defenses. TNF-α activates phagocyte functions and facilitates granuloma formation and maintenance, and patients with autoimmune or chronic inflammatory diseases undergoing anti-TNF-α therapies have increased susceptibility for reactivation of latent tuberculosis or infection with nontuberculous mycobacteria (NTMs) (1, 2, 46, 47). IL-6 modulates the immune response to mycobacteria and has various effects dependent on the nature of the challenge; mouse infection models have inconsistently shown that IL-6 signaling is protective, favors, or does not significantly affect M. tuberculosis or M. avium infection (48–52). How this translates to humans is not yet clear, as few data exist regarding the risk of mycobacterial infections in patients receiving anti-IL-6 treatment (1).

Upon infection, IRF1 is first activated by PRR signaling and then sustained via autocrine/paracrine TNF-α and IL-6. In uninfected bystander macrophages, IRF1 activation is most likely driven by cytokines, although we cannot exclude the possibility that soluble PRR ligands are shed from M. avium and contribute to the activation. IRF1 is involved in regulating several antimicrobial activities, such as the expression of inducible nitric oxide synthase (iNOS/NOS2) (11), guanylate binding proteins (GBPs) (19, 38), and IRG1 (22) and regulation of tissue granulomas during mycobacterial infection (9, 12), in addition to basal antiviral activity (53). Many of these are initiated by interferon signaling and studied in whole-animal infection models or IFN-γ-primed macrophages, which is less relevant for the present work since we did not detect significantly increased interferon production from M. avium-infected human primary macrophages. For instance, iNOS-driven production of NO is shown to be an important part of antimycobacterial defenses at the organismal level and in murine macrophages, whereas human macrophages do not produce a fulminant NO response, at least not *in vitro* (54–56). We thus chose to focus on IRG1 and confirm previous reports that the expression is under the partial control of IRF1. Further, our findings show that IRG1 contributes to the control of M. avium infection both in primary human and in mouse macrophages, most plausibly through the production of itaconate, with inhibition of M. avium isocitrate lyases, methylcitrate lyase, or methylmalonyl-coenzyme A (CoA) mutase, the metabolic rewiring needed for growth when intraphagosomal M. avium relies on fatty acids as a carbon source (23, 41). However, to our surprise, only low levels of itaconate could be detected in M. avium-infected macrophages despite significant IRG1 induction. Several mechanisms may explain this observation and have been explored herein.

One possibility is that M. avium consumes itaconate. Itaconate catabolic pathways have been described in other bacteria, such as *Yersinia*, Pseudomonas, and M. tuberculosis (57, 58). Orthologous genes are found in M. avium; however, since PFA-killed M. avium induced even less itaconate production than live bacteria, the low levels in infected macrophages cannot be explained by itaconate degradation or dissimilation.

A second possibility is that itaconate is directly consumed by the host macrophage. As with the microbial pathway, itaconate can be metabolized into itaconyl-CoA and, via citramalyl-CoA, into acetyl-CoA and pyruvate by the enzyme CLYBL in mammalian cells (59). However, itaconate produced in macrophages activated by LPS or other TLR ligands is readily detectable at high levels, including in our experiments. Moreover, Ruetz and colleagues proposed a direct antimycobacterial activity of itaconyl-CoA against M. tuberculosis by scavenging the essential vitamin B_12_, adding to the effect of itaconate itself (41).

Finally, it may be that, despite overall low levels of endogenous itaconate in M. avium-infected macrophages, higher concentrations are present in phagosomes either by local production or directed delivery. In an elegant study by Chen et al., the authors expressed an itaconate biosensor in Salmonella based upon an “itaconate operon” and showed that itaconate was efficiently delivered to the SCV through intimate contact with mitochondria (25). We failed to visualize if IRG1 was present in the vicinity of M. avium phagosomes, as neither of the two commercially available antibodies tested worked for immunofluorescence. We did, however, observe mitochondria in close apposition to M. avium phagosomes, suggesting that directed delivery of itaconate is possible. Moreover, Chen et al. estimated the concentration of itaconate in the SCVs to be approximately 6 mM (25), which should be sufficient to impair bacterial growth also for M. avium. Our results show that 1 to 10 mM exogenously provided itaconate is required for growth inhibition of M. avium in culture and inside macrophages. However, the microbicidal activity of itaconate is most likely dependent on pH, since neutralization made it less efficient in inhibiting M. avium growth in culture. This was also recently suggested by others (42, 43). There are also conflicting reports with regard to the membrane permeability of itaconate, and several derivatives have been made to facilitate cellular uptake (30, 60). Based on work of Swain and colleagues which shows that itaconate can cross the cell membrane, we assume, but did not determine, that both itaconate and 4-OI were taken up by the human macrophages (30). Neutralization should make itaconate less membrane permeable and thus reduce the cellular uptake, but this was necessary since high concentrations (>100 μM) of unadjusted itaconate acidified the medium to a level that was toxic to the macrophages. However, intraphagosomal bacteria are subject to other forms of stress and are in a metabolic state different from that of bacteria grown in rich culture medium. Thus, lower concentrations of itaconate than what is needed to inhibit growth in culture may inhibit M. avium growth intracellularly, or as suggested by Chen et al. (25) using Salmonella, directed delivery of itaconate to mycobacterial compartments may reach microbicidal concentrations.

Another possible caveat is that itaconate derivatives like 4-OI are not necessarily metabolized into itaconate when entering the cell (30), and their strong electrophilic properties can induce immunoregulatory effects that are different from those of unmodified itaconate. Both itaconate and its derivatives have proposed immunoregulatory activities linking metabolism and inflammation, such as the inhibition of select inflammatory cytokines (26–32), induction of tolerance (32), and induction of the Nrf2-driven electrophilic stress response program (26–28, 30). However, an anti-inflammatory or tolerant state should facilitate intracellular M. avium growth, similar to what is observed when inflammatory cytokines (TNF-α, IL-6 [present study]) and NF-κB signaling (6) are blocked or in knockout mice (4, 39), which is opposite to the antimycobacterial activity that we observe when IRG1 is activated or exogenous itaconate is provided. We also explored another possible explanation: that IRG1 mediates bactericidal activity by regulating beta-oxidation-dependent mitochondrial ROS (mROS) production (24). However, inhibition of beta-oxidation using etomoxir did not impact the growth of M. avium in macrophages, suggesting that an alternative function of IRG1 in controlling mROS does not explain the antimycobacterial effects of IRG1 in our system. Combined, our results thus favor a model derived from the study by Chen et al. (25), in which mitochondrial IRG1 can mediate targeted delivery of itaconate to M. avium phagosomes at sufficient concentrations for antibacterial activity. However, this model needs to be verified experimentally.

Taking our findings together, we describe a protective mechanism whereby M. avium, directly via TLRs and indirectly via autocrine/paracrine TNF-α and IL-6 signaling, activates IRF1-driven expression of IRG1 in infected and uninfected bystander macrophages. IRG1 has antimycobacterial effects by preventing intracellular growth, possibly through directed delivery of itaconate to M. avium phagosomes (Fig. 7B), although alternative unknown antimycobacterial functions of IRG1 cannot be excluded. This mechanism contributes to the understanding of why patients on anti-inflammatory treatment, e.g., with tocilizumab or infliximab, can be more susceptible to mycobacterial disease.

## MATERIALS AND METHODS

### Reagents.

The following antibodies were purchased from the indicated suppliers: IRF1 (Santa Cruz; C20), IRG1 (ThermoFisher; PA5-102893 and Abcam ab222411), LAMP1 (Santa Cruz; H4A3), and TOMM20 (Sigma; HPA011562). Alexa Fluor 555-conjugated goat anti-rabbit and Hoechst 33342 dye were from Life Technologies. Ultrapure LPS (E. coli 0111:B4), TLR2 ligand FSL-1, and TLR8 ligand CL-75 were purchased from InvivoGen. Tocilizumab and infliximab were obtained from Roche and Pfizer, respectively. The JAK I inhibitor (CAS 457081-03-7) was obtained from Sigma. Itaconate and 4-octyl itaconate were obtained from Sigma.

### Isolation and differentiation of human primary macrophages.

Buffy coats from healthy blood donors were provided by the blood bank at St. Olav’s Hospital, Trondheim, Norway, after we obtained informed consent and the approval of the Regional Committee for Medical and Health Research Ethics (no. 2009/2245). Peripheral blood mononuclear cells (PBMCs) were isolated using density gradient centrifugation (Lymphoprep; Axis-Shield Point of Care). Monocyte-derived macrophages (MDMs) were generated by plastic adherence for 1 h in complete RPMI 1640 (680 μM l-glutamine and 10 mM HEPES; Gibco) supplemented with 5% pooled human serum (the blood bank, St. Olav’s Hospital) at 37°C and 5% CO_2_. After three washing steps with Hanks’ balanced salt solution (Gibco), monocytes were cultivated for 6 days with a change of medium at day 3 to RPMI 1640–10% human serum and 10 ng/ml recombinant macrophage colony-stimulating factor (M-CSF; R&D Systems). At day 6, the medium was replaced with RPMI 1640–10% human serum and used for experiments on day 7.

### siRNA transfection of MDMs.

Transfection with siRNA was performed using siLentFect lipid reagent (Bio-Rad) for RNA interference (RNAi) according to the manufacturer’s protocol. Gene knockdown was evaluated by reverse transcription-quantitative PCR (RT-qPCR). Pooled IRF1 and IRG1 ON-TARGETplus human siRNAs (Dharmacon/Thermo Scientific) were used to target *IRF1* and *IRG1*. MDMs were treated with 20 nM siRNA two times (day −4 and day −2) before the medium was changed to fresh medium (RPMI 1640–10% human serum), and then they were allowed to rest for 1 to 2 h and challenged with TLR ligands or M. avium.

### RNA extraction and RT-qPCR analysis of mRNA levels.

MDMs were washed with cold phosphate-buffered saline (PBS) and lysed in buffer RLT (Qiagen) with 1% β-mercaptoethanol. Total RNA was extracted using an RNeasy minikit and QIAcube according to the manufacturer’s protocol (Qiagen), which included DNase I digestion (RNase-free DNase set). The samples included in the study presented a ratio of the optical density at 260 nm (OD_260_) to the OD_280_ of ∼2, assessed using an ND-1000 spectrophotometer (NanoDrop). cDNA was synthetized from normalized amounts of RNA using the high-capacity RNA-to-cDNA kit according to the manufacturer’s recommendations (Applied Biosystems). qPCRs were performed in a 20-μl total volume with a 10-ng cDNA input by the PerfeCta qPCR FastMix, UNG, ROX (Quanta Biosciences), and TaqMan (Applied Biosystems) gene expression assays with the following: glyceraldehyde-3-phosphate dehydrogenase (GAPDH; Hs99999905_m1), TNF-α (Hs00174128_m1), IL-6 (Hs00985639_m1), IFN-α (Hs00265051_s1), IFN-β (Hs01077958_s1), IFN-γ (Hs00989291_m1), IRF1 (Hs00971960_m1), and IRG1 (Hs00985781_m1). The targeted genes were amplified with a StepOnePlus real-time PCR system, and relative quantities of gene expression were calculated using the comparative CT method, with GAPDH gene expression as an endogenous control.

### M. avium culture, macrophage infection, and challenge with TLR ligands.

M. avium clone 104 expressing cyan fluorescent protein (CFP) or DsRed was cultured in liquid Middlebrook 7H9 medium (Difco/Becton, Dickinson) supplemented with 0.5% glycerol, 0.05% Tween 80, and 10% albumin dextrose catalase. In some experiments, 0.5% acetate was added to the medium in place of glycerol. Cultures were maintained at log-phase growth (optical densities were between 0.3 and 0.6 and measured at 600 nm [OD_600_]) in a 180-rpm shaking incubator at 37°C for a maximum of 5 days. On the day of infection, bacteria were washed with PBS, sonicated, and passed through a gauge 15 needle to ensure a single-cell suspension before we challenged day 7 MDMs for 10 min at a multiplicity of infection of 10. MDMs were subsequently washed and maintained in culture for the appropriate time. In some experiments, the following MDMs were challenged for 4 h with TLR ligands at the indicated concentrations: FSL-1 (TLR2; 50 ng/ml), ultrapure LPS (TLR4; 100 ng/ml), and CL75 (TLR8; 4 and 0.8 μM, which correspond to 500 and 100 ng/ml).

### Immuno-staining.

Human MDMs cultivated on glass-bottomed 96-well plates (IBL) were fixed and permeabilized using a standard protocol as previously described (7). Briefly, cells were fixed in 4% PFA for 10 min and then incubated in NH_4_Cl for 10 min to quench PFA-induced auto-fluorescence prior to permeabilization with PBS–0.05% saponin. Cells were next incubated for at least 90 min in PBS–0.05% saponin–20% human serum to reduce nonspecific binding before being stained with primary antibodies (1 μg/ml) in PBS–0.05% saponin–1% human serum overnight at 4°C. Cells were washed with PBS–0.05% saponin–1% human serum and incubated with secondary antibodies for 45 min at room temperature, washed again, and stored at 4°C in PBS containing Hoechst dye for nuclear staining.

### mRNA fluorescence *in situ* hybridization.

Fluorescence *in situ* hybridization was conducted using the RNAscope multiplex fluorescence V2 kit (ACD Bio, Bio-Techne) according to manufacturer recommendations. Briefly, MDMs were cultivated on a glass-bottomed 96-well plate and infected with M. avium-CFP for 5 h and 24 h. After fixation with 4% PFA, cells were digested for 10 min with a 1/15 dilution of the protease solution. mRNA was stained using an Alexa Fluor 488-labeled probe (IL-6), a Cy3-labeled probe (TNF-α), and a Cy5-labeled probe (IRG1) with the appropriate dilution. The signal of each probe was specifically amplified using a Trypticase soy agar (TSA) superamplification kit (Perkin Elmer), with TSA diluted at 1/1,000.

### Imaging.

MDMs cultivated on glass-bottomed 96-well plates were imaged with a Zeiss LSM880 confocal microscope with a 20× numerical aperture (NA) objective of 0.8 or a 63× NA of 1.4 (Carl Zeiss Micro-imaging Inc.). Emissions were collected using GaAsP hybrid detectors. The following acquisition parameters were used: a numerical zoom set to 0.6 and frame averaging set to 1, with three-dimensional (3D) acquisition to collect the entire cell with a z step of 0.75 μm. Each fluorophore was recorded using sequential acquisition to minimize cross excitation and channel bleed-through. Hoechst dye was excited with a 405-nm diode laser, and emission was collected through a 420- to 440-nm window. CFP was excited with a 458-nm argon laser, and emissions were collected through a 470- to 500-nm window. Alexa 488 was excited with a 488-nm argon laser, and emissions were collected through a 505- to 550-nm window. DsRed was excited with 543-nm HeNe lasers, and emissions were collected through a 560- to 610-nm window. Cy5 was excited with a 633-diode laser, and emissions were collected through a 645- to 700-nm window. Images were analyzed with Image J (NIH). Images taken with the 63× objective were subjected to deconvolution using Huygens Professional (Scientific Volume Imaging) with the “confocal low signal” setting.

### *In situ* CFU measurement and mRNA hybridization quantification.

3D stacks were projected using the “sum” setting. Resulting images were converted to 8 bits. Regions of interest were drawn around macrophages containing M. avium. The background was estimated using HiLo lookup tables and subtracted. A minimum of 250 infected cells per condition and per donor were counted.

### Cytokine measurements.

Supernatants from human MDMs challenged with M. avium were collected, and cytokine secretion profiles for TNF-α, IL-6, IL-18, IL-23, IL-1α/β, IL-8, IP-10, MCP-1, GRO-α, MIP-1α/β, SDF1-α, IL-10, IL-1RA, and IFN-α/β were analyzed according to the manufacturer's instructions using the ProcartaPlex human cytokine and chemokine panel (Affymetrix, eBioscience).

### Mass spectrometric quantification of intracellular itaconate levels.

Sampling and extraction for mass spectrometric quantification of intracellular itaconate levels were performed from 5 to 8 million MDMs, as described for adherent cell lines in reference 61. Absolute quantification was performed by tandem mass spectrometry (MS/MS) coupled with two different chromatographic separation techniques for all extracts to allow validation of measured concentrations close to the noise levels (Fig. S5). Lyophilized extracts were reconstituted in MilliQ-H_2_O for a capillary ion chromatography (capIC)-MS/MS-based analysis, performed as described in reference 62 with the modifications described in reference 63. A second, liquid chromatography (LC)-based analysis was performed with an Acquity I-class ultrahigh-performance liquid chromatograph (UPLC) coupled with a Xevo TQ-XS triple-quadrupole mass spectrometer equipped with an electrospray source operating in negative mode, with application of an LC protocol adapted from reference 64. For LC-MS/MS, lyophilized extracts were reconstituted in 1/4 (vol/vol) H_2_O-acetonitrile (ACN) and injected (5 μl) onto a SeQuant ZIC-pHILIC 100- by 2.1-mm column with a pore size of 5 μm (150462; Merck). The column was maintained at 45°C and eluted with filtered (0.45-μm) mobile phases A, 3/2 (vol/vol) H_2_O-ACN, and B, 1/9 (vol/vol) H_2_O/ACN, both added to 10 mM ammonium acetate at pH 9. The following gradient was applied at a flow rate of 200 μl/min: 0 to 1 min, 80% B; 1 to 15 min, 80 to 1% B; 15 to 15.5 min, 1 to 80% B; and 15.5 to 20 min, 80% B. Itaconate was quantified from the precursor product ion transition, as follows: *m/z*, 129 to 85; the coefficient of variation [CV], 30 V; and the coefficient error (CE), 8 eV. Data processing was performed in the TargetLynx application manager of MassLynx 4.1 (Waters). Absolute quantification was performed by interpolation of a calibration curve prepared from serial dilutions of an itaconate standard (I29204; Sigma-Aldrich). The calibration curve was calculated by least-squares regression with 1/*x* weighting. Response factors of the analytical standard and biological extracts were corrected by the corresponding response factor of the U-^13^C-labeled isotopologue of itaconate (SC-495554; Santa Cruz Biotechnology) spiked into the samples. Extract concentrations were normalized to seeding density and are reported as macrophage cell volumes (65) to obtain intracellular concentrations.

### Statistical analysis.

Normality was tested for each experiment. A two-tailed *t* test and analysis of variance (ANOVA) test were used on normally distributed data; the Mann-Whitney test was used otherwise. Areas under the curve were calculated for each cytokine/donor couple. Significant *P* values were set as follows: <0.05 (*), <0.01 (**), and <0.001 (***). Statistical analyses were performed using GraphPad Prism 8 (GraphPad Software, Inc., San Diego, CA, USA).
