# Supplementary figures and images for: The Tumor Necrosis Factor Alpha and Interleukin 6 Auto-paracrine Signaling Loop Controls Mycobacterium avium Infection via Induction of IRF1/IRG1 in Human Primary Macrophages
Source: mBio. 2021 Oct 5;12(5):e02121-21. doi: 10.1128/mBio.02121-21 (PMC8546851; doi:10.1128/mBio.02121-21)

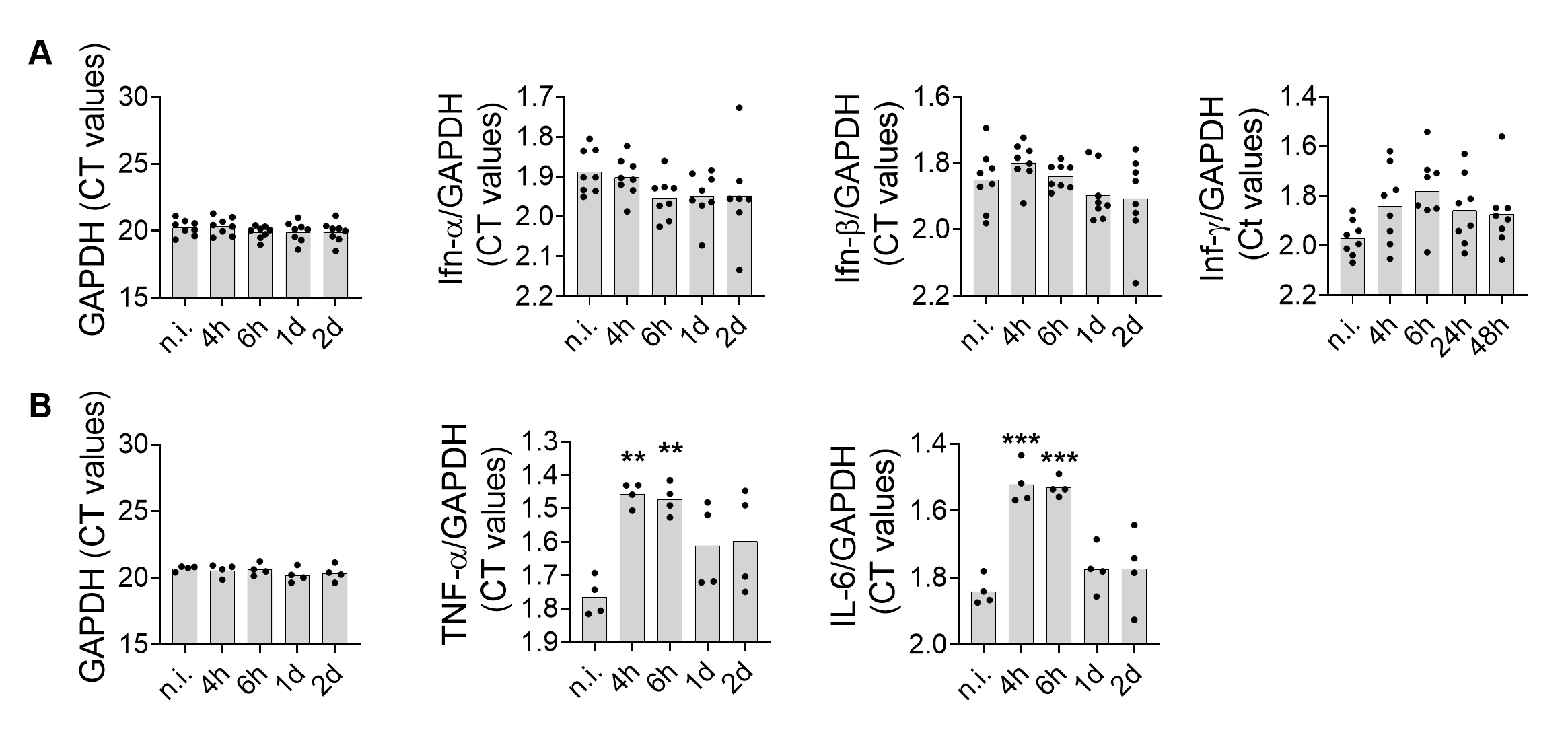

Supplement: FIG S2 [file mbio.02121-21-sf002.tif]
